# Progesterone has rapid positive feedback actions on LH release but fails to reduce LH pulse frequency within 12 h in estradiol‐pretreated women

**DOI:** 10.14814/phy2.12891

**Published:** 2016-08-17

**Authors:** Eleanor G. Hutchens, Katherine A. Ramsey, Louisa C. Howard, Michelle Y. Abshire, James T. Patrie, Christopher R. McCartney

**Affiliations:** ^1^ Division of Endocrinology and MetabolismDepartment of MedicineUniversity of Virginia School of MedicineCharlottesvilleVirginia; ^2^ Center for Research in ReproductionUniversity of Virginia School of MedicineCharlottesvilleVirginia; ^3^ University of Virginia School of MedicineCharlottesvilleVirginia; ^4^ College and Graduate School of Arts and SciencesUniversity of VirginiaCharlottesvilleVirginia; ^5^ Department of Public Health SciencesUniversity of Virginia School of MedicineCharlottesvilleVirginia

**Keywords:** Circadian rhythm, estradiol, gonadotropin surge, gonadotropin‐releasing hormone, luteinizing hormone, luteinizing hormone surge, progesterone

## Abstract

In women, progesterone suppresses luteinizing hormone (LH) (gonadotropin‐releasing hormone) pulse frequency, but how rapidly this occurs is unknown. In estradiol‐pretreated women in the late follicular phase, progesterone administration at 1800 did not slow sleep‐associated LH pulse frequency. However, mechanisms controlling LH pulse frequency may differ according to sleep status; and we thus hypothesized that progesterone acutely suppresses waking LH pulse frequency. This was a randomized, double‐blind, crossover study of LH secretory responses to progesterone versus placebo administered at 0600. We studied 12 normal women in the late follicular phase (cycle days 7–11), pretreated with 3 days of transdermal estradiol (0.2 mg/day). Subjects underwent a 24‐h blood sampling protocol (starting at 2000) and received either 100 mg oral micronized progesterone or placebo at 0600. In a subsequent menstrual cycle, subjects underwent an identical protocol except that oral progesterone was exchanged for placebo or vice versa. Changes in 10‐h LH pulse frequency were similar between progesterone and placebo. However, mean LH, LH pulse amplitude, and mean follicle‐stimulating hormone exhibited significantly greater increases with progesterone. Compared to our previous study (progesterone administered at 1800), progesterone administration at 0600 was associated with a similar increase in mean LH, but a less pronounced increase in LH pulse amplitude. We conclude that, in estradiol‐pretreated women in the late follicular phase, a single dose of progesterone does not suppress waking LH pulse frequency within 12 h, but it acutely amplifies mean LH and LH pulse amplitude – an effect that may be influenced by sleep status and/or time of day.

## Introduction

Gonadotropin‐releasing hormone (GnRH) is secreted in a pulsatile fashion from a functionally coordinated collection of hypothalamic neurons. Variations in GnRH pulse frequency stimulate differential synthesis and secretion of luteinizing hormone (LH) and follicle‐stimulating hormone (FSH) from the pituitary, with high and low frequencies favoring LH and FSH secretion respectively (Wildt et al. 1981; Gross et al. 1987). Progesterone is a major negative feedback regulator of GnRH pulse frequency in adult women. However, the rapidity with which progesterone slows LH (and by inference GnRH) pulse frequency in women remains unclear. Although studies in cows and sheep suggest that progesterone reduces GnRH pulse frequency within 2–6 h (Bergfeld et al. 1996; Skinner et al. 1998; Robinson et al. 2000), corresponding data in humans are mixed: some studies suggest rapid slowing (within 8–14 h) (Minakami et al. 1984; Permezel et al. 1989), while others suggest that more prolonged progesterone exposure is required (Pastor et al. 1998; McCartney et al. 2007a).

The rapidity with which progesterone suppresses LH pulse frequency is of particular relevance to our previously proposed hypotheses regarding day‐night GnRH pulse frequency regulation in early pubertal girls: in particular, we previously proposed that the early morning increase in progesterone concentration rapidly (within hours) inhibits GnRH pulse frequency, contributing to daytime slowing in early puberty (Blank et al. 2006; McCartney et al. 2007b, 2009). Given challenges inherent to research in early pubertal girls (e.g., blood withdrawal limits), we initially sought to interrogate a key component of this hypothesis – namely, that progesterone can acutely reduce LH pulse frequency – via a study in which eight normal, estradiol‐pretreated women received progesterone or placebo at 1800 during the latter half of the follicular phase (McCartney et al. 2007a). Compared to placebo, progesterone did not suppress LH pulse frequency within 12 h.

Although the aforementioned study in adults suggested that progesterone does not rapidly suppress LH pulse frequency in women, postprogesterone (and postplacebo) assessments were performed primarily during sleep periods. We subsequently reported a small, nonrandomized study of early pubertal girls suggesting that suppression of daytime pulses (but not nighttime pulses) occurs within 3–7 h of oral progesterone administration (Collins et al. 2012). Differential control of GnRH pulse frequency based on sleep status is also consistent with studies suggesting that dietary calorie restriction slows daytime, but not nighttime, LH pulse frequency in women studied during the late follicular phase (Loucks and Heath 1994; Loucks et al. 1998). Therefore, given that sleep could have been a confounder in our earlier adult study (McCartney et al. 2007a), the current study was designed to test the *primary hypothesis* that morning (0600) administration of progesterone rapidly (within 12 h) suppresses *waking* (1000–2000) LH pulse frequency in normal, estradiol‐pretreated adult women studied during the late follicular phase (cycle days 7–11).

In our earlier study (McCartney et al. 2007a), LH pulse amplitude, mean LH, and mean FSH markedly increased within 4 h of progesterone (but not placebo) administration. These findings reflected progesterone positive feedback, a previously described phenomenon that may contribute to the midcycle gonadotropin surge (Hoff et al. 1983; Liu and Yen 1983; Terasawa et al. 1987). Of interest, some studies in women (Cahill et al. 1998; Kerdelhue et al. 2002), but not all (Hoff et al. 1983), suggest that the LH surge is most likely to be initiated in the early morning hours, implying that circadian rhythms may play a role in this regard. Accordingly, as a prespecified analysis, we tested a *secondary hypothesis* that early morning (0600) administration of progesterone (current study) provokes greater increases in LH pulse amplitude and mean LH compared to evening (1800) administration of progesterone (previous study McCartney et al. 2007a).

## Materials and Methods

The Institutional Review Board at the University of Virginia approved all study procedures, which were in accordance with the ethical standards of the Helsinki Declaration of 1975, as revised in 2008. The study was registered with ClinicalTrials.gov (identifier NCT00594217).

### Subjects

Twelve healthy, nonobese women with regular menstrual cycles and no evidence of hyperandrogenism completed the study and were included in the analysis (Table 1). Self‐identified race was Asian (4 subjects), Black (1 subject), Hispanic (1 subject), Middle‐Eastern (1 subject), and White (5 subjects). None of the subjects reported previous pregnancy, recent weight loss, or excessive exercise. Study participants had taken no medications known to affect the reproductive axis in the 90 days prior to study.

**Table 1:** Subject characteristics. The number of subjects is 12 for all variables. To convert metric units to SI units: total testosterone (ng/dL) ×0.0347 (nmol/L); free testosterone (pg/mL) ×3.467 (pmol/L); insulin (μIU/mL) ×7.175 (pmol/L); glucose (mg/dL) ×0.0555 (mmol/L)

	Mean	SD	Median	Range
Age (years)	20.6	3.9	19	18–31
Body mass index (kg/m^2^)	22.4	3.1	21.72	18.4–28.5
Body fat percentage (%)	25.0	6.7	23.3	16.4–37.8
Waist‐to‐hip ratio	0.76	0.05	0.76	0.67–0.84
Total testosterone (ng/dL)	15.8	7.7	14.14	5.1–32.2
SHBG (nmol/L)	40.1	14.3	36.85	19.5–67.1
Free testosterone (pg/mL)	2.6	1.3	2.3	0.7–5.9
Fasting insulin (μIU/mL)	4.0	3.0	2.4	<2–12.2
Fasting glucose (mg/dL)	84	4	85.5	77–88

SHBG, sex hormone binding globulin.

### Study procedures

Full written informed consent was obtained from each study participant. Each volunteer underwent detailed history, physical exam, and laboratory testing to screen for hormonal and health‐related abnormalities, as previously described (McCartney et al. 2007a). All participants had normal screening laboratory tests.

The study followed a crossover design, with assessment of the acute effects of progesterone and placebo (individually) on pulsatile LH secretion for each subject. Subjects were randomized to receive either progesterone or placebo during the first overnight admission, with the second overnight study occurring during a subsequent menstrual cycle. Researchers and subjects were blinded to the order of progesterone administration.

Subjects received transdermal estradiol patches (0.1 mg/day/patch, 2 patches [delivering a total of 0.2 mg/day] on the abdomen and changed every 2 days) starting between cycle *days 4 and 8* (inclusive) and continued for a total of 4 days, with overnight admission occurring on day 3 of estradiol administration. Exogenous estradiol was given to standardize hypothalamic exposure to estradiol and to help ensure sufficient hypothalamic progesterone receptor density (MacLusky and McEwen 1978; Romano et al. 1989; Scott et al. 2000). Immediately prior to estradiol initiation, subjects had blood drawn for progesterone and *β*‐hCG to help exclude a luteal phase (progesterone <1.0 ng/dL) and pregnancy respectively.

On day 3 of estradiol administration (i.e., cycle *day 7–11*), participants were admitted to the Clinical Research Unit (CRU) at 1800. Beginning at 2000, blood for later hormone measurement was obtained through an indwelling intravenous catheter over a 24‐h period as follows: LH every 10 min; progesterone every 30 min; FSH, estradiol, and testosterone every 2 h. Sex hormone binding globulin, fasting insulin, and glucose were measured at 0600. After 10 h of sampling (i.e., at 0600), either oral micronized progesterone (100 mg) suspension or oral placebo suspension was administered according to randomization.

Standard meals were given at standard times during CRU admissions, and subjects fasted between 2200 and 0600. Subjects were encouraged to sleep from 2200 to 0600, but subjects were otherwise not allowed to sleep (i.e., before 2200 or after 0600). Sleep periods were assessed throughout the admission using wrist actigraphy (Sadeh and Acebo 2002). Estradiol patches were removed at the end of the 24‐h sampling period; and volunteers were discharged on oral iron supplementation (325 mg twice daily) to help replenish iron stores.

During a subsequent cycle, subjects underwent a second CRU admission identical to the first (including pretreatment with estradiol) except that oral progesterone was exchanged for placebo or *vice versa* according to the crossover study design.

### Hormonal measurements

All hormone assays were performed by the Ligand Assay and Analysis Core of the Center for Research in Reproduction (University of Virginia) as previously described (McCartney et al. 2007a). All samples from an individual woman were analyzed in duplicate in the same assay for each hormone. LH, FSH, and progesterone were measured by chemiluminescence (sensitivities 0.1, 0.1 IU/L, 0.2 ng/mL; intra‐assay coefficients of variation [CVs] 3.3%, 3.2%, 4.4%; and interassay CVs 5.8%, 4.9%, 7.8%, respectively; Siemens Healthcare Diagnostics, Los Angeles, CA). Total testosterone was measured by radioimmunoassay (sensitivity 10 ng/dL, intra‐assay CV 4.3%, interassay CV 7.4%; Calbiotech, Spring Valley, CA). Estradiol was measured by radioimmunoassay (sensitivity 10 pg/mL, intra‐assay CV 6.3%, interassay CV 8.1%; American Laboratory Products Company, Salem, NH). Samples with measured values below assay sensitivity were assigned the value of the assay's sensitivity. To convert from conventional to Systeme International (SI) units: progesterone ×3.18 (nmol/L); total testosterone ×3.47 (pmol/L); estradiol ×3.671 (pmol/L).

### Data analysis

Given that LH measurement error is unequal across the physiological range of LH concentrations (heteroscedastic), a computerized data reduction protocol (*StdCurve*, developed by Michael L. Johnson, Ph.D., University of Virginia) was employed to provide a variance model for experimental measurement error for each LH concentration time series (i.e., for each subject admission). *StdCurve* utilizes (1) unprocessed and untruncated luminometer output data in addition to (2) corresponding LH readout data from the standard curve for all LH measurements in the assay run, including blanks (no LH) and known standards (i.e., quality controls approximating LH concentrations of 2.5, 25, and 60 IU/L). In essence, *StdCurve* assigns statistically accurate estimates of LH concentration precision for each time point across the LH time series, eliminating truncation errors introduced at low LH concentrations by the proprietary standard curve. Experimental error is addressed by assigning and propagating uncertainty estimates for each standard curve response (including zero dose responses) by an empirically determined discrete uncertainty profile. These discrete uncertainty profiles account for both response precision (replicability) and accuracy (deviation from the predicted calibration curve) without relying on assumed theoretical variance‐assay response relationships.

For each LH concentration time series, pulsatile secretion was identified and characterized using *AutoDecon*, a multiparameter deconvolution program (Johnson et al. 2008). This statistically based algorithm is fully automated in that it identifies initial parameter estimates while simultaneously performing deconvolution. In particular, *AutoDecon* iteratively inserts and tests the significance of presumed secretion events. The automated nature of *AutoDecon* renders it nonsubjective, in contrast with earlier deconvolution procedures. Compared to *Cluster 7* (Veldhuis and Johnson 1986), *AutoDecon* has greater sensitivity to detect LH pulses (96% vs. 80%), but a higher false positive rate (6% vs. 1%) (Johnson et al. 2008). To limit false positives, only the following *AutoDecon*‐identified pulses were included in subsequent analyses: (1) pulses with at least two peak values that were at least 10% higher than the nadir value; or (2) pulses with at least one peak value that was at least 20% higher than the nadir value.

Once pulse locations were established, LH pulse frequency was estimated by calculating the average interpulse interval (IPI) over time blocks using a method described in detail previously (McCartney et al. 2007a). Briefly, the number of minutes within the time block was divided by the number of LH IPIs (including fractions thereof) residing in that time block. LH pulse amplitudes were calculated by subtracting the preceding LH nadir concentration from the peak LH concentration for each pulse.

### Power analysis, subject dropout, and final sample size

The primary endpoint for this study was the change in LH pulse frequency (average IPI over 10 h) attributable to progesterone. We hypothesized that 10‐h IPI after progesterone administration (1000–2000) would be higher than 10‐h IPI after placebo administration (i.e., that progesterone would reduce LH pulse frequency). Based on the within‐subject variability in LH IPI differences (progesterone vs. placebo) observed in our earlier study (McCartney et al. 2007a), we estimated that the study of 12 women would provide 80% power to detect a 16.7‐min difference (progesterone vs. placebo) in average 10‐h IPI.

Formal study procedures were initiated in 16 women. For various personal reasons, 3 women elected to drop out of the study after completing a single admission only; data from these subjects are not included in this analysis. Thirteen women completed the full study protocol. However, one of these women was not included in the analysis because she had an average progesterone concentration of 4.8 ng/mL from 2000 to 0600 *before* receiving exogenous progesterone, indicating that she was inadvertently studied during the luteal phase. Thus, we formally analyzed the data for 12 women who completed both admissions during the late follicular phase.

### Statistical analysis

The influence of exogenous progesterone on LH IPI was analyzed using a 2‐period crossover design analysis of covariance (ANCOVA) – our primary endpoint analysis. In the ANCOVA model specification, the interblock changes (i.e., the 10‐h time block after progesterone/placebo [1000–2000] minus the 10‐h time block before progesterone/placebo [2000–0600]) in log_e_ LH IPI represented the response data. (For this and subsequent analyses, log_e_ transformation was necessary to ensure conformity with the normality assumptions of ANCOVA procedures.) The ANCOVA model factors were sequential order of interventions (i.e. progesterone followed by placebo vs. placebo followed by progesterone), crossover period (i.e., first vs. second), and intervention (progesterone vs. placebo). Baseline log_e_ LH IPI (i.e., during the 2000–0600 time block) served as an ANCOVA adjustment variable. With regard to hypothesis testing, our primary hypothesis was that the change in mean log_e_ LH IPI from the baseline state (2000–0600) to the post‐treatment state (1000–2000) was the same irrespective of whether progesterone or placebo was administered at 0600. The primary hypothesis was tested based on a linear contrast of the ANCOVA least‐squared means, and a two‐sided *P* ≤ 0.05 decision rule was established a priori as the null hypothesis rejection rule. Traditional residual diagnostics were used to confirm model goodness of fit.

With regard to secondary endpoint variables, mean LH, LH pulse amplitude, mean FSH, and progesterone were analyzed via 2‐period crossover ANCOVA in exactly the same manner as the LH IPI data. Since estradiol and testosterone concentrations were not hypothesized to change in response to progesterone administration, they were analyzed as log_e_ total concentrations over each 24‐h admission via mixed‐effect ANCOVA models with “subject” as a random effect (i.e., blocking factor) and the intervention (progesterone, placebo) as the fixed effect factor.

As a prespecified secondary analysis, we compared the influence of progesterone given at 0600 (current study) versus 1800 (previous study McCartney et al. 2007a) on mean LH concentration and mean LH pulse amplitude. We hypothesized that, compared to progesterone administered at 1800, progesterone administered at 0600 would provoke greater increases in both mean LH and LH pulse amplitude. To maximize the validity of these comparisons, we reanalyzed the data published in 2007 (McCartney et al. 2007a) using *StdCurve* and *AutoDecon*, as described above. Since this secondary analysis involved multiple simultaneous comparisons, Bonferroni correction was employed to limit experimental‐wise type I error to ≤0.05.

Although the above outcome data were log_e_ transformed prior to analysis, we report geometric means (GM) and associated 95% confidence intervals (CI) when describing the results of statistical testing. The GM is a location parameter similar to arithmetic mean, calculated as the antilog of the mean of log‐transformed values. Thus, GM provides a faithful representation of the data as analyzed (in contrast to arithmetic mean). Another benefit is that GM ratios can be interpreted as percentage and/or fold changes.

Of potential importance, one subject in the current cohort had mean LH and LH pulse amplitude responses that were markedly discordant with the other 11 subjects. We suspected, but could not confirm, that the samples for her progesterone and placebo admissions had been mislabeled prior to assay. For this reason, we repeated the aforementioned analyses after excluding this subject. We do not report the results of this secondary analysis, however, because exclusion of this subject did not alter the results of our primary analysis (LH pulse frequency), and it only strengthened apparent differences between treatment conditions for mean LH, LH pulse amplitude, and mean FSH.

The statistical software package SAS version 9.4 (SAS Institute Inc., Cary, NC) was used to conduct all statistical analyses.

## Results

Progesterone admissions occurred on cycle day 9.7 ± 1.4 (mean ± SD), and placebo admissions on cycle day 9.8 ± 1.3. As measured by wrist actigraphy, sleep periods (time from first sleep to last sleep) were 7.6 ± 0.4 and 7.1 ± 0.9 h – and sleep efficiency 92 ± 4 and 92 ± 12% – during the progesterone and placebo admissions respectively. As dictated by simple (i.e., nonblocked) randomization, progesterone was given during the first admission in three subjects and placebo in nine; for all ANCOVA analyses, sequence order was not a significant predictor of results.

### Sex steroids

**Figure 1:**
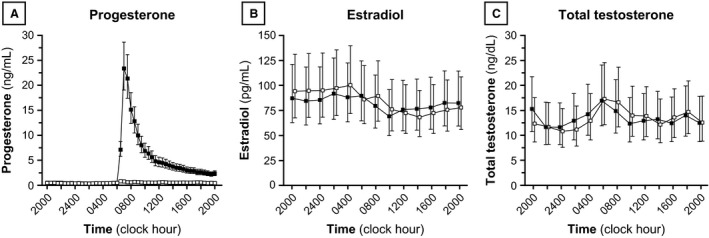
Progesterone (A), estradiol (B), and total testosterone (C) concentrations. Each data point identifies the geometric mean (GM), and vertical lines identify 95% confidence intervals for the GM. Progesterone and placebo admissions are denoted by solid and open data points, respectively. Conversion from metric to Systeme International units: progesterone ×3.18 (nmol/L); estradiol ×3.67 (pmol/L); total testosterone ×0.0347 (nmol/L).

Summary statistics for progesterone, estradiol, and total testosterone concentrations are shown in Table 2, and sex steroid profiles are represented graphically in Figure 1. Ten‐hour progesterone concentrations at baseline (2000–0600) were similar between the progesterone and placebo admissions (GM ratio 1.05; 95% CI: [0.75–1.48], *P* = 0.746). As expected, 10‐h (1000–2000) progesterone concentrations increased markedly after progesterone administration (9.4‐fold increase in GM; 95% CI: [7.71–11.48], *P* < 0.001). After placebo administration, 10‐h progesterone concentration increased slightly compared to baseline (27% increase in GM; 95% CI: [4–55%], *P* = 0.021). The increase associated with progesterone administration was 7.64‐fold greater than the increase associated with placebo administration (95% CI: [6.21–9.41], *P* < 0.001).

**Table 2:** Summary statistics, sex steroid concentrations. Summary statistics for progesterone are partitioned by admission (progesterone vs. placebo) and time block (2000–0600 [preintervention] vs. 1000–2000 [postintervention]). Summary statistics for estradiol and total testosterone are for the entire 24‐h admission and, thus, partitioned by admission only. The number of subjects is 12 for all variables. To convert metric units to SI units: progesterone ×3.18 (nmol/L); estradiol ×3.67 (pmol/L); total testosterone ×0.0347 (nmol/L)

	Admission	Time block (h)	Mean	SD	GM	Median	Range
Progesterone (ng/mL)	Progesterone	2000–0600	0.48	0.15	0.45	0.5	0.2–0.8
		1000–2000	3.96	1.35	3.77	3.7	2.4–7.1
	Placebo	2000–0600	0.43	0.09	0.42	0.4	0.3–0.6
		1000–2000	0.53	0.12	0.52	0.55	0.4–0.8
Estradiol (pg/mL)	Progesterone	2000–2000	98.8	60.8	83.2	76.5	28–224
	Placebo	2000–2000	92.9	34.1	87.1	81.5	38–162
Testosterone (ng/dL)	Progesterone	2000–2000	15.5	6.5	14.2	14.7	6.1–28.0
	Placebo	2000–2000	16.1	7.4	14.6	14.2	5.1–32.3

GM, geometric mean.

Estradiol levels were similar during both admissions: GM 83.2 pg/mL during the progesterone admission and 87.1 pg/mL during the placebo admission (GM ratio between admissions 0.96; 95% CI: [0.71–1.29], *P* = 0.746). Total testosterone levels were also similar between admissions (GM ratio 0.98; 95% CI: [0.84–1.12], *P* = 0.751).

### LH pulse frequency

**Figure 2:**
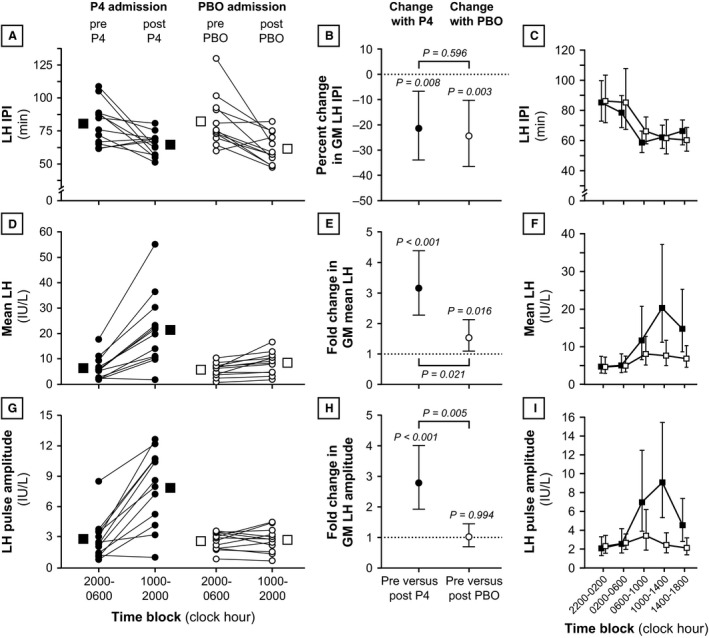
Luteinizing hormone (LH) secretory characteristics during progesterone and placebo admissions. Rows illustrate data for LH interpulse interval (panels A, B, and C), mean LH (panels D, E, and F), and LH pulse amplitude (panels G, H, and I). Panels A, D, and G: The first column illustrates data for 10‐h time blocks immediately before intervention (2000–0600) versus 10‐h time blocks after intervention (1000–2000). Individual data are represented by connected circles, and arithmetic mean values are shown as larger squares. Progesterone and placebo admissions are denoted by solid and open data points, respectively. Panels B, E, and H: The second column illustrates changes pre versus post‐intervention (i.e., 2000–0600 vs. 1000–2000). The points identify the percentage change (B) or fold change (E and H) in the geometric mean (GM), and the vertical lines identify the 95% confidence interval for the percentage or fold change. *P* values above individual data plots relate to pre versus postintervention changes *within an individual admission*. *P* values connecting admissions relate to *between‐admission comparisons* of pre versus postintervention changes (i.e., pre vs. postprogesterone change vs. pre vs. postplacebo change). Panels G, H, and I: The third column illustrates data for two 4‐h time blocks before intervention and three 4‐h time blocks after intervention. Each data point identifies the GM, and vertical lines identify 95% confidence intervals for the GM. Progesterone and placebo admissions are denoted by solid and open data points, respectively. Note that statistical analyses were not performed for 4‐h time blocks. IPI, interpulse interval; P4, progesterone; PBO, placebo.

Summary statistics for LH IPI are shown in Table 3, and results are represented graphically in Figure 2. Ten‐hour GM LH IPI decreased from 79.0 min during the first 10‐h time block (2000–0600, before progesterone administration) to 64.0 min during the second 10‐h time block (2000–0600, after progesterone administration) – a 21% decrease in GM IPI (95% CI: [7–34%], *P* = 0.008). Similar changes occurred during the placebo admission: GM LH IPI decreased from 80.3 min before placebo to 60.5 min after placebo, representing a 24% GM IPI reduction (95% CI: [10–36%], *P* = 0.003). After adjusting for GM IPI during the first 10‐h time block – values of which were similar between progesterone and placebo admissions (*P* = 0.913) – changes in GM IPI from first to second 10‐h time block were not different between the progesterone and placebo admissions (ratio of changes, 1.05; 95% CI: [0.87–1.27], *P* = 0.596).

**Table 3:** Summary statistics, gonadotropin characteristics. Summary statistics are partitioned by admission (progesterone vs. placebo) and time block (2000–0600 [preintervention] vs. 1000–2000 [postintervention]). The number of subjects is 12 for all variables

	Admission	Time block	Mean	SD	GM	Median	Range
LH IPI (min)	Progesterone	2000–0600	80.4	16.1	79.0	80.3	61.4–108.7
		1000–2000	64.5	8.9	64.0	65.1	51.1–80.7
	Placebo	2000–0600	82.1	19.3	80.3	75.1	59.7–129.9
		1000–2000	61.5	11.6	60.5	57.9	47.3–82.0
Mean LH (IU/L)	Progesterone	2000–0600	6.3	4.6	5.0	5.5	1.8–17.6
		1000–2000	21.3	14.3	16.4	20.7	1.8–54.9
	Placebo	2000–0600	5.7	2.7	4.9	6.3	0.9–10.3
		1000–2000	8.4	4.3	7.2	8.8	1.9–16.5
LH amplitude (IU/L)	Progesterone	2000–0600	2.8	2.1	2.2	2.4	0.7–8.5
		1000–2000	7.8	3.8	6.5	8.2	1.0–12.6
	Placebo	2000–0600	2.5	0.9	2.4	2.8	0.8–3.6
		1000–2000	2.7	1.2	2.4	2.8	0.6–4.4
Mean FSH (IU/L)	Progesterone	2000–0600	4.1	1.6	3.8	4.7	1.5–5.9
		1000–2000	8.0	3.3	7.4	8.0	3.0–14.6
	Placebo	2000–0600	3.5	1.0	3.3	3.5	1.4–5.4
		1000–2000	4.7	1.7	4.4	4.5	2.0–7.0

### Mean LH and LH pulse amplitude

Ten‐hour mean LH during the first 10‐h time block was similar before progesterone and placebo (GM 5.0 and 4.9 IU/L, respectively; *P* = 0.985) (Table 3 and Fig. 2). After progesterone administration, mean LH increased markedly (GM 16.4 IU/L) – an estimated 3.15‐fold increase in GM versus baseline (95% CI: [2.26–4.39], *P* < 0.001). Mean LH also increased after placebo administration (1.52‐fold estimated increase in GM; 95% CI [1.09–2.13], *P* = 0.016). After adjusting for mean LH in the first 10‐h time block, pre versus postintervention changes in GM mean LH (first vs. second 10‐h time blocks) were 2.07‐fold greater with progesterone compared to placebo (95% CI: [1.15–3.72], *P* = 0.021).

Luteinizing hormone pulse amplitudes were similar in the 10 h before progesterone and placebo administration (GM 2.2 and 2.4 IU/L, respectively; *P* = 0.417) (Table 3 and Fig. 2). Ten‐hour GM LH amplitude increased to 6.5 IU/L after progesterone, an estimated 2.78‐fold increase (95% CI: [1.92–4.01], *P* < 0.001); but GM LH amplitude was unchanged after placebo (GM 2.4 IU/L; *P* = 0.994). Intervention‐related changes in GM LH pulse amplitude were 2.68‐fold greater with progesterone compared to placebo (95% CI: [1.46–4.92], *P* = 0.005).

### Mean FSH

**Figure 3:**
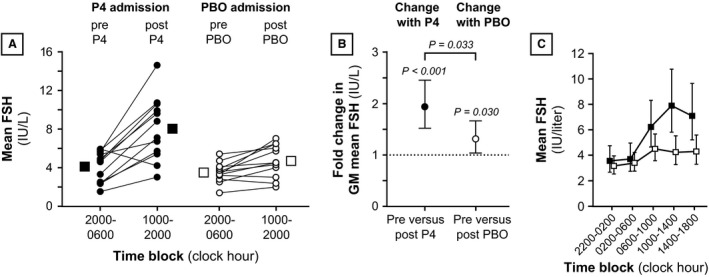
Mean follicle‐stimulating hormone (FSH) during progesterone and placebo admissions. Panel A: Data for 10‐h time blocks immediately before intervention (2000–0600) versus 10‐h time blocks after intervention (1000–2000). Panel B: Pre versus postintervention changes (i.e., 2000–0600 vs. 1000–2000). Panel C: Data for two 4‐h time blocks before intervention and three 4‐h time blocks after intervention. All data are organized as in Figure 2. P4, progesterone; PBO, placebo.

Ten‐hour FSH concentrations at baseline were similar between the progesterone and placebo admissions (GM 3.8 and 3.3 IU/L, respectively; *P* = 0.842) (Table 3 and Fig. 3). Ten‐hour FSH increased after progesterone administration (1.93‐fold estimated increase in GM FSH; 95% CI: [1.52–2.46], *P* < 0.001); after placebo, 10‐h mean FSH increased to a lesser degree (an estimated 31% increase in GM FSH compared to baseline; 95% CI: [3–66%], *P* = 0.030). Intervention‐related changes in GM FSH were 1.49‐fold higher with progesterone compared to placebo (95% CI: [1.04–2.13], *P* = 0.033).

### Influence of time of day on changes in mean LH and LH pulse amplitude

**Figure 4:**
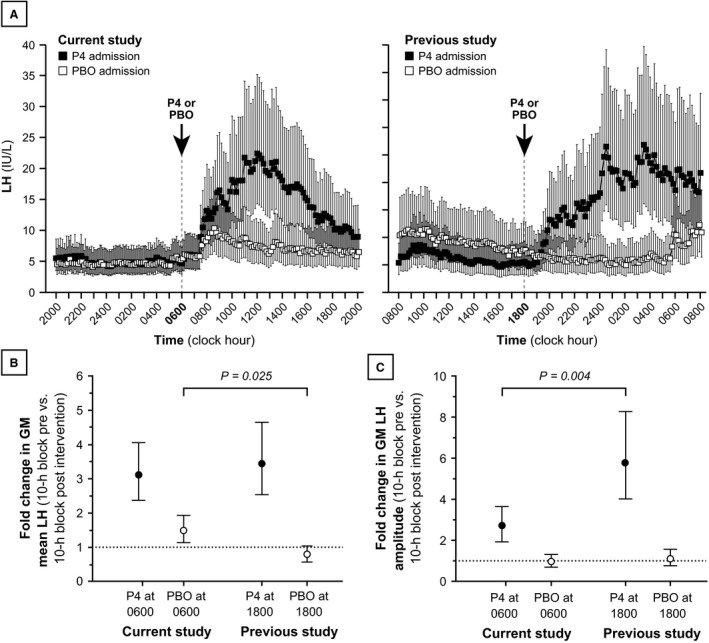
Mean luteinizing hormone (LH) and LH pulse amplitude during progesterone and placebo admissions for the current study and for our previous study (McCartney et al. 2007a). Panel A: LH concentrations (measured every 10 min) for the current study (left) and the previous study (right). Progesterone and placebo admissions are denoted by solid and open data points, respectively. Each data point identifies the geometric mean (GM), and vertical lines identify 95% confidence intervals for the GM. Panels B and C: Fold changes in mean LH (B) and LH pulse amplitude (C), pre versus postintervention. The specific changes shown are as follows: 2000–0600 versus 1000–2000 when progesterone or placebo was administered at 0600; and 0800–1800 versus 2200–0800 when progesterone or placebo was administered at 1800. The points identify the fold change in the GM, and the vertical lines identify the 95% confidence interval for the fold change. *P* values (Bonferroni‐corrected) relate to *between‐study comparisons* of pre versus postintervention changes, ostensibly reflecting clock hour differences (e.g., pre vs. postintervention change when progesterone was given at 0600 vs. pre vs. postintervention change when progesterone was given at 1800). P4, progesterone; PBO, placebo.

Compared to our previous study in which progesterone was administered in the evening (1800) (McCartney et al. 2007a), progesterone administered at 0600 (current study) produced similar increases in mean LH (Fig. 4). In the previous study, 10‐h GM mean LH increased 3.43‐fold (95% CI: [2.53–4.65], *P* < 0.001) from the first 10‐h time block (0800–1800) to the second (2200–0800) – similar to the increase observed in the current study (difference between studies, Bonferroni‐corrected *P* = 1.00). Ten‐hour GM mean LH pre versus postplacebo did not change significantly in the previous study (*P* = 0.073), while 10‐h GM LH increased between the first and second 10‐h time blocks when placebo was given at 0600 (described above). Changes in GM mean LH associated with placebo administration was 1.73‐fold greater (95% CI: [1.16–2.58]) in the current study compared to the previous study (Bonferroni‐corrected *P* = 0.025).

When progesterone was administered in our previous study, GM LH amplitude increased 5.76‐fold (95% CI: [4.01–8.27]) between the first and second 10‐h time blocks (*P* < 0.001); but 10‐h GM LH amplitude did not change pre versus postplacebo administration (*P* = 0.749). The progesterone‐associated increase in GM LH amplitude was 52% lower (95% CI: [26–68%]) in the current study (progesterone given at 0600) compared to the previous study (progesterone given at 1800) (between‐study difference in progesterone‐associated changes, Bonferroni‐corrected *P* = 0.004). Between the two studies, the placebo admissions were associated with similar changes in GM LH amplitude (Bonferroni‐corrected *P* = 0.879).

### Post hoc assessments

**Figure 5:**
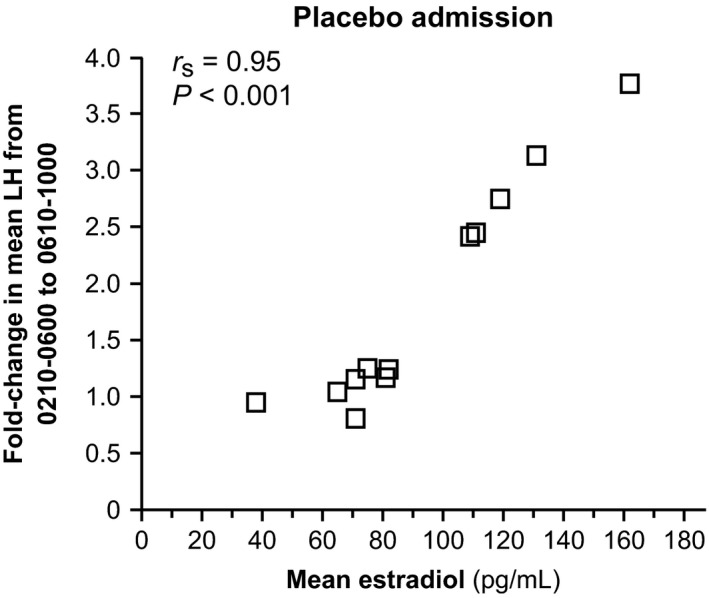
Relationship between average estradiol concentrations and sleep–wake changes in mean LH during placebo admissions. Subjects were awakened at 0600, and the sleep–wake change in mean LH (*y*‐axis) is shown as the fold‐change in mean LH from 0210–0600 to 0610–1000. Conversion from metric to Systeme International units: estradiol ×3.67 (pmol/L).

Although not part of prespecified analyses, we noted that mean LH frequently increased – sometimes markedly so – within 4 h of waking during the placebo admission of the current study, as implied in Figure 4A. In particular, subjects were awakened at 0600, and 4‐h mean LH increased from 0210–0600 to 0610–1000 in 10 of 12 placebo admissions, with an average fold increase of 1.8. Five subjects with marked (ranging from 2.4‐ to 3.8‐fold) increases in mean LH within 4 h of waking had estradiol concentrations over 100 pg/mL, while the seven subjects without obvious changes in mean LH within 4 h of waking (ranging from a 24% decrease to a 25% increase) had estradiol concentrations lower than 100 pg/mL. Accordingly, average estradiol concentrations during the placebo admission positively correlated with both absolute change in mean LH (Spearman correlation: *r*_s_ = 0.84, *P* < 0.001) and the fold‐change in mean LH (*r*_s_ = 0.95, *P* < 0.001) within 4 h of waking (Fig. 5). Similar relationships were observed in the progesterone admissions – absolute and fold mean LH changes from 0210–0600 to 0610–1000 correlated with estradiol (for both: *r*_s_ = 0.70, *P* = 0.011) – similar to a previous study (Permezel et al. 1989).

Changes in LH pulse amplitude within 4 h of waking during placebo admissions were less consistent, with LH pulse amplitude increasing in six of 12 subjects. The overall average increase was 61%, largely driven by four subjects – all with estradiol concentrations >100 pg/mL – who exhibited 2.2‐ to 3.7‐fold increases. However, estradiol concentrations did not clearly correlate with absolute or fold‐changes in LH pulse amplitude within 4 h of waking during placebo admissions (*r*_s_ 0.44–0.47, *P* > 0.1 for both) or during progesterone admissions (for both: *r*_s_ = 0.42, *P* = 0.18).

We noted that achieved estradiol levels were highly variable. Variable absorption (e.g., variable patch adherence to the skin) could have played a role in this regard, as suggested by within‐subject (i.e., between‐admission) differences, which were as follows (pg/mL): mean ± SD, 33 ± 33; 25th percentile, 4.5; median, 24; 75th percentile, 50.5. Cycle day did not correlate with achieved estradiol level (*r*_s_ = 0.004, *P* = 0.985, *n* = 24 admissions). In addition, average subject estradiol (i.e., average of two admissions) did not correlate with age, BMI, or waist‐to‐hip ratio (*P* > 0.1 for all, *n* = 12 subjects). While average subject estradiol was negatively correlated with body fat percentage (*r*_s_ = −0.64, *P* = 0.026 [unadjusted for multiple comparisons]), achieved estradiol concentration did not correlate with body fat percentage when evaluating (1) the progesterone admissions in isolation or (2) the placebo admissions in isolation (*P* > 0.2 for both). Lastly, it remains possible that other (unknown) factors might explain the strong correlation between achieved serum estradiol concentration and the morning rise in mean LH during placebo admissions; but morning increases in LH did not correlate with age, BMI, body fat percentage, or waist‐to‐hip ratio.

## Discussion

Although our previous study failed to disclose a rapid reduction in sleep‐related LH pulse frequency when a single dose of progesterone was administered at 1800 (McCartney et al. 2007a), we considered the possibility that the regulation of GnRH pulse frequency differs according to sleep status – a possibility suggested by a study in early pubertal girls (Collins et al. 2012) and other studies in late follicular phase women (Loucks and Heath 1994; Loucks et al. 1998). We thus hypothesized that progesterone administration at 0600 slows waking LH pulse frequency within 12 h in normal adult women pretreated with estradiol in the late follicular phase. Results from our current study do not support this hypothesis; however, in keeping with earlier findings (McCartney et al. 2007a), we observed (1) sleep‐related reductions in LH pulse frequency, and (2) rapid and marked increases in gonadotropin concentrations (both LH and FSH) and LH pulse amplitude associated with progesterone administration. Our confidence in these findings is enhanced by methodological improvements employed with the current study: in particular, our larger study population (*n* = 12 vs. 8) provided greater statistical power; we employed a more sophisticated LH pulse analysis protocol (e.g., *StdCurve* plus *AutoDecon* vs. *Cluster*); and we subjected the data to more sophisticated statistical analyses.

The rapidity with which progesterone slows GnRH pulse frequency in women remains unclear. Although a study in postmenopausal women suggested that high dose IM progesterone – to achieve concentrations approximating 20–30 ng/mL – at 0600 suppressed LH pulse frequency by 45% within 9–14 h (Minakami et al. 1984), this study involved a markedly different patient population (older, estrogen‐deficient subjects) and a number of methodological differences, including the absence of a control state (e.g., untreated group). A study in normally cycling women studied on either cycle day 6 or 10 (*n* = 4 each) suggested that 10 mg intramuscular progesterone – achieving mean progesterone concentrations of 1.6 ng/mL – reduced LH pulse frequency within 8 h (i.e., the ratio of postprogesterone IPI to preprogesterone IPI was 1.24, *P* = 0.05) (Permezel et al. 1989). However, in a similar group of 16 women, LH pulse frequency was not acutely altered by mifepristone administration (Permezel et al. 1989). In another study, progesterone (50 mg every 8 h by vaginal suppository) and estradiol (0.2 mg/day by transdermal patch) – both started in the late follicular phase (cycle day 8–10) and achieving progesterone concentrations circa 9–10 ng/mL – appeared to reduce LH pulse frequency by 50% within 5 days (Pastor et al. 1998); but this change was not statistically significant, perhaps a reflection of the small number of subjects (*n* = 5) and limited statistical power.

The rapidity with which progesterone slows GnRH pulse frequency in human females has been of particular interest to our group, in large part because we have previously proposed a hypothesis of day‐night LH pulse frequency regulation in early puberty that was predicated on the notion that progesterone can have rapid effects (within hours) on LH pulse frequency – specifically, we proposed that the early morning increase in progesterone rapidly (within hours) suppresses daytime LH pulse frequency, thus contributing to day**–**night differences in LH pulse frequency (Blank et al. 2006; McCartney et al. 2007b, 2009). Although our current study does not support the hypothesis that progesterone rapidly inhibits LH pulse frequency in women, the dynamics of progesterone suppression may vary according to hormonal milieu. For example, androgens appear to antagonize the negative feedback effects of progesterone on GnRH neuron firing rates in rats (Pielecka et al. 2006) and on LH pulse frequency in both sheep (Robinson et al. 1999) and women (Pastor et al. 1998; Eagleson et al. 2000). Therefore, when compared to feedback dynamics in adult women, progesterone‐mediated suppression may be more rapid – in addition to being more profound – when androgen concentrations are very low (e.g., in normal early puberty). This consideration may help explain the apparent discordance between our current study and the aforementioned study in early pubertal girls, which suggested a rapid suppression (within 4–7 h) of waking LH pulse frequency after progesterone administration (Collins et al. 2012).

Some published data suggest that LH surge initiation tends to be initiated in the early morning hours (Cahill et al. 1998; Kerdelhue et al. 2002). Since LH surge initiation is largely a reflection of sex steroid positive feedback on pituitary LH release, we tested a predefined secondary hypothesis: progesterone positive feedback on mean LH and LH pulse amplitude would be more pronounced when administered in the morning (0600; current study) compared to evening administration (1800; previous study McCartney et al. 2007a). Progesterone‐mediated changes in mean LH were similar regardless of the timing of progesterone administration, consistent with a previous report in monkeys (Terasawa et al. 1984). In contrast, placebo‐associated changes were different: mean LH increased after placebo administered at 0600, but decreased after placebo administered at 1800. We infer that this latter difference could reflect diurnal and/or sleep–wake changes in mean LH (discussed further below).

With regard to LH pulse amplitude, progesterone‐associated increases were greater with evening (1800) dosing compared to morning (0600) dosing – in contrast with our hypothesis. We considered the possibility that this particular finding reflected interstudy differences in LH pulse frequency, since LH pulse amplitude tends to increase as a function of previous IPI (O'Dea et al. 1989). That is, since LH IPI decreased from the first to second 10‐h time blocks in the current study, one might have expected a concomitant decrease in LH pulse amplitude; and since LH IPI increased from the first to second 10‐h time blocks in the previous study, one might have expected a concomitant increase in LH pulse amplitude. However, such changes in LH pulse amplitude were not clearly observed during the placebo admissions (Fig. 4C). An alternative explanation for greater progesterone‐related increases in LH pulse amplitude in the previous study is that, for unclear reasons, those subjects had higher average estradiol levels (214 ± 46 [mean ± SD] and 169 ± 23 pg/mL during the progesterone and placebo admissions, respectively) despite identical dose and duration of transdermal estradiol use. We speculate that, compared to the current study, higher estradiol levels in the previous study augmented the positive feedback responses to progesterone.

We noted that mean LH commonly increased – sometimes markedly so – within 4 h of waking (at 0600) during the placebo admission in the current study; such changes correlated with ambient E2 concentrations. Mean LH tended to be highest in the early morning hours (circa 0600–1000) in our earlier study as well (see Fig. 4). As described above, some (but not all) studies in women suggest that LH surges tend to be initiated in the morning. For example, one study of 19 ovulatory women suggested that LH surges were initiated in the early morning hours (circa 0400–0800) (Kerdelhue et al. 2002). Given that average estradiol concentrations positively correlated with changes in mean LH within 4 h of waking, we speculate that this could represent nascent surge‐like activity – specifically associated with time of day and/or the transition from sleep to wake – that that will be further amplified as preovulatory estradiol (and progesterone) concentrations are achieved.

## Conclusion

Herein, we provide data suggesting that daytime/waking LH pulse frequency is not suppressed within 12 h by a single 100 mg oral dose of progesterone in estradiol‐pretreated women studied in the late follicular phase. However, mean LH and LH pulse amplitude are acutely amplified by progesterone in this setting. Our data suggest that progesterone‐mediated augmentation of LH pulse amplitude may be affected by time of day and/or sleep status – being more prominent in the evening/nighttime hours compared to the morning/daytime hours – although differences in achieved estradiol concentrations represent an important potential confounder in this regard.

## Conflicts of Interest

The authors have no conflicts of interest to disclose.
